# The Safety and Suitability of DNA Sequencing of Tissue Biopsies Performed on Patients Referred to a Phase I Unit

**DOI:** 10.3390/cancers16244252

**Published:** 2024-12-20

**Authors:** Angela Esposito, Edoardo Crimini, Carmen Criscitiello, Carmen Belli, Roberta Scafetta, Raimondo Scalia, Grazia Castellano, Elisa Giordano, Jalissa Katrini, Liliana Ascione, Luca Boscolo Bielo, Matteo Repetto, Antonio Marra, Dario Trapani, Gianluca Maria Varano, Daniele Maiettini, Paolo Della Vigna, Franco Orsi, Elena Guerini Rocco, Nicola Fusco, Giuseppe Curigliano

**Affiliations:** 1 Division of Early Drug Development, European Institute of Oncology, IRCCS, 20141 Milan, Italy; angela.esposito@ieo.it (A.E.); edoardo.crimini@ieo.it (E.C.);; 2 Department of Oncology and Hemato-Oncology, University of Milan, 20122 Milan, Italy; 3 Department of Medical Oncology, Campus Bio-Medico University of Rome, 000128 Rome, Italy; 4 Section of Medical Oncology, Department of Surgical, Oncological and Oral Sciences, University of Palermo, 90127 Palermo, Italy; 5 Division of Interventional Radiology, European Institute of Oncology, IRCCS, 20141 Milan, Italy; 6 Division of Pathology, European Institute of Oncology, IRCCS, 20141 Milan, Italy

**Keywords:** targeted therapies, precision oncology, phase I trials, biopsy, next-generation sequencing, cancer, agnostic therapy, biomarker, real world, drug development

## Abstract

Background: Early-phase clinical trials offer a unique opportunity for patients with cancer. These trials often mandate biopsies to collect tumor tissue for research purposes, requiring patients to undergo invasive procedures. Some trials mandate molecular prescreening, but the success of these analyses relies on the quality and quantity of the tested materials. Additionally, bioptic procedures may result in complications. Methods: We retrospectively examined the records of patients referred to the Early Drug Development (EDD) Unit of the European Institute of Oncology who underwent biopsies for research purposes between January 2014 and December 2022. Our objective was to assess the safety of biopsy procedures and adequacy of the samples for NGS testing. Results: In total, 355 out of 731 patients (48.6%) underwent protocol-mandated biopsies. The most frequent sites of biopsy were the liver, lymph nodes, skin, and breast. Histological diagnosis was achieved in 349 (98%) patients, and NGS testing was successfully conducted in 111/127 (88.4%) cases. Of the 16 unsuccessful NGS attempts, 9 were performed on liver tissue. Unsuccessful NGS testing was attributed to poor sample quality and/or quantity, and the success rate varied significantly based on the specific tests attempted. Complications occurred in a small proportion of patients (4.8%), and none were serious. Conclusions: The non-negligible failure rate of NGS testing highlights the crucial need for implementing specific guidelines and Standard Operating Procedures for samples intended for NGS. With the use of a risk-based biopsy framework to guide clinical decisions, procedure-related complications may be minimized.

## Simple Summary

Early-phase clinical trials often require new biopsies for research purposes, which can be affected by complications. Samples need to meet quantitative and qualitative standards to be adequate for next-generation sequencing (NGS) testing. We aimed to assess the safety and adequacy of NGS for research tissue biopsies. The biopsies were found to be safe, with only a small proportion of patients experiencing mild adverse events (4.8%). The NGS success rate was 88.4% and varied significantly according to the specific panel attempted.

## 1. Introduction

Cancer stands as a leading cause of death globally [1]. Despite significant advancements in the types, number, and efficacy of treatments for cancer, there remains a pressing need for innovative drugs that can enhance survival outcomes for cancer patients [2,3]. In this context, early-phase clinical trials offer patients an opportunity to access new treatments from their first development, from the bench to the bedside [4,5]. In recent years, the landscape of early drug development in oncology has undergone substantial evolution. Advances in molecular biology, immunotherapy, and the elucidation of the complete sequence of the human genome have led to the development of highly specific targeted therapies tailored to different types of cancers. Phase I protocols now increasingly involve the preliminary evaluation of anti-tumor activity and the targets of new drugs and are not only intended to inform dose finding regardless of patient-specific outcomes. Many of these trials incorporate non-diagnostic biopsies aimed at understanding the biologic effects of pharmacologic interventions and also informing patient selection. However, non-diagnostic tissue collection is associated with significant costs and may potentially dissuade patients from participating in early-phase clinical trials, especially when these have no impact on the treatment choice and do not provide improved therapeutic delivery. To address ethical concerns and enhance oversight, the American Society of Clinical Oncology (ASCO) has introduced a comprehensive and risk-based framework for research biopsies, aiming to maximize scientific utility and minimize harm for patients [6]. The invasiveness of biopsies can be a concern for patients, leading to potential enrollment refusals [5,7]. While these biopsies often provide proof of mechanism and help define the optimal biologic dose, they may not directly impact patient outcomes. However, in some cases, biopsies are used to perform next-generation sequencing (NGS) tests with therapeutic intent, revealing actionable molecular alterations [8,9,10,11,12]. The identification of actionable alterations through NGS opens the theoretical possibility of administering targeted therapies, which have been shown to improve survival outcomes across various cancer histologies [13,14,15,16,17,18]. However, strict requirements for NGS testing must be met, including histological material quantity and quality; otherwise, the test may fail [19].

In this article, we present our institutional experience with patients undergoing newly collected biopsies for enrollment in early-phase clinical trials. Our objective was to assess the safety of biopsies, providing insights for clinicians during discussions with patients about trial enrollment. Moreover, we aimed to evaluate the frequency with which these biopsies yield adequate material for histological diagnosis and NGS testing.

## 2. Materials and Methods

We conducted a retrospective review of electronic medical records of patients referred to the Early Drug Development (EDD) Unit of the European Institute of Oncology (IEO) in Milan. This review encompassed individuals who underwent biopsies for research purposes from January 2014 to December 2022. The biopsies were performed when required according to the protocol of each clinical trial, and their feasibility and safety were first discussed with the physician in charge of performing the procedure.

Data were extracted from the IEO EDD prospective database. This database is Excel-based and contains information on medical history, performed procedures, treatments, and adverse events, structured for clinical trial data collection purposes. Adverse events were codified according to the Common Terminology Criteria for Adverse Events (CTCAE) v5, as consistently documented for all patients in our medical records. Pseudonymized patients were selected based on enrollment in a trial within the EDD Unit and having undergone a biopsy for study inclusion. Patients who underwent NGS testing on the biopsy and had available results were included in the analysis of adequacy for NGS. NGS was performed using multiple panels and platforms, as required by clinical trial protocols. All NGS procedures were conducted on Formalin-Fixed Paraffin-Embedded [FFPE] tissue specimens. This study was approved by the local internal review board (number UID 3560).

The standard requirements for histological diagnosis varied depending on the sampling site and the histology of the neoplasm. For the purposes of this study, specimens were deemed inadequate for histological diagnosis based on the pathologist’s report. Similarly, the adequacy for NGS testing was assessed based on the test report or communications with the test provider. An unsuccessful NGS test was defined as either rejection of the sample or failure of the test. Samples deemed inadequate for histological diagnosis were not sent for NGS testing. If NGS testing failed, a new sample from the same histological specimen was sent, depending on the availability of additional material. If the second attempt was successful, it was classified as adequate for NGS testing.

Statistical analyses were conducted using R v 4.3.2 (R Development Core Team, free license). The Chi-square test of independence was used to assess significant relationships between two categorical variables, while Fisher’s exact test was used for categorical variables when the expected value in one of the contingency tables was less than 5. A *p* value < 0.05 was considered statistically significant. All tests were two-tailed.

## 3. Results

### 3.1. Characteristics of Patients and Biopsies

We identified a total of 731 patients referred to the EDD Unit and enrolled in clinical trials. Of these, 355 patients (48.6%) underwent a new tissue biopsy at trial entry, as required by protocol. Among this group, the median age was 56 years (range: 20–83), and the majority (79%) were female. The most common tumor type was breast cancer (53.5%), followed by cutaneous melanoma (6.8%) and carcinoma of unknown primary (5.8%). Table 1 provides a list of the clinicopathological characteristics of patients who underwent tissue biopsies.

The most frequent biopsy sites were the liver (148, 41.7%), lymph nodes (64, 18%), skin (46, 13.1%), and breast (27, 7.3%). All the liver and lymph node percutaneous needle biopsies were ultrasound (US)-guided, while lung and bone biopsies were computed tomography (CT)-guided. Breast biopsies were primarily US-guided. The majority of patients underwent percutaneous needle biopsies (276 patients, 77.7%). The most commonly used needle size was 18 Gauge (221/276, 80%), followed by 16 Gauge (15, 5.4%), 14 Gauge (13, 4.7%), and 12 Gauge (2, 0.7%); needle size was unspecified for the remaining 25 patients. Sixty-three patients (17.7%) underwent punch biopsies, eight underwent endoscopic biopsies, and eight underwent surgical procedures to obtain histological material. The median time from signing informed consent for the trial to biopsy was 3 days (range: 0–83).

### 3.2. Adequacy of Biopsies for Histological Diagnosis and NGS

**Figure 1:**
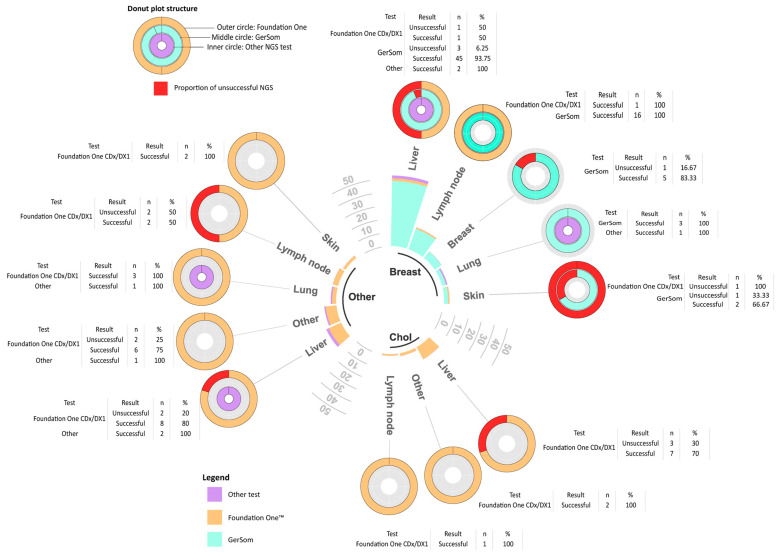
Circular grouped and stacked barplot: bars represent the sites of biopsy, grouped by primary tumors (showed inside the circle: Breast, breast cancer; Chol, cholangiocarcinoma; Other, other tumor types). Only tumors that counted at least 5 NGS attempts are reported separately from “Other”. The height of the bars corresponds to the number of biopsies performed at the anatomic site in the specific tumor type. Bars of stacked colors represent the number of each NGS test (see legend). Donut plots report the success rate of each NGS test for the biopsy site and primary tumor. The inner, middle, and outer circles represent other tests, GerSom and Foundation One^TM^, respectively. The color of each test is consistent with the circular barplot. The proportion of failures for each test is reported in red. The tables provide the corresponding data.

Tumor tissue was generally adequate for histological diagnosis, with a success rate of 98% (349/355). Samples that were inadequate for histological diagnosis due to the absence of a tumor component were not considered suitable for an NGS testing attempt. For 168 patients, no information was available on the results of the analyses conducted on the tissue, as the trial sponsor mandated biopsies but did not provide reports to the investigators. A total of 139 patients underwent NGS testing on the study biopsy, and for all of them, the molecular report was accessible to the investigators. For the remaining 48 patients, biomarkers were tested with methods other than NGS (e.g., Polymerase Chain Reaction [PCR], Immunohistochemistry (IHC), or In Situ Hybridization [ISH]). NGS was successful in 111 (87.4%) of the 127 cases with available NGS results. Nine of the sixteen unsuccessful NGS tests were performed on liver tissue; however, there was no statistically significant difference in the rate of NGS failures in liver samples compared to other biopsy sites (*p* = 0.861). Similarly, NGS failure was not associated with the primary tumor histotype (*p* = 1). Seventy-six patients underwent NGS testing within the SHARP phase 2 clinical trial (EudraCT number 2017-003216-39; IEO 674), a monocentric academic trial that employed the GerSom panel, a custom amplicon-based assay testing 467 genes. In contrast, 44 patients underwent testing with the Foundation One™ CDx or DX1 test, and 7 patients underwent other tests. Interestingly, we found a statistically significant difference in the inadequacy rate among Foundation One™ (11/44 tests, 25%) and GerSom (5/76 tests, 6.6%; *p* = 0.004). Due to the small number of patients who underwent other heterogeneous genomic tests, we did not perform a correlation analysis between their failure rates and those of the Foundation One^tm^ and GerSom panels. Figure 1 provides a comprehensive representation of the biopsy sites grouped by the primary tumor, along with the corresponding NGS tests performed and the rate of failures.

### 3.3. Safety

**Figure 2:**
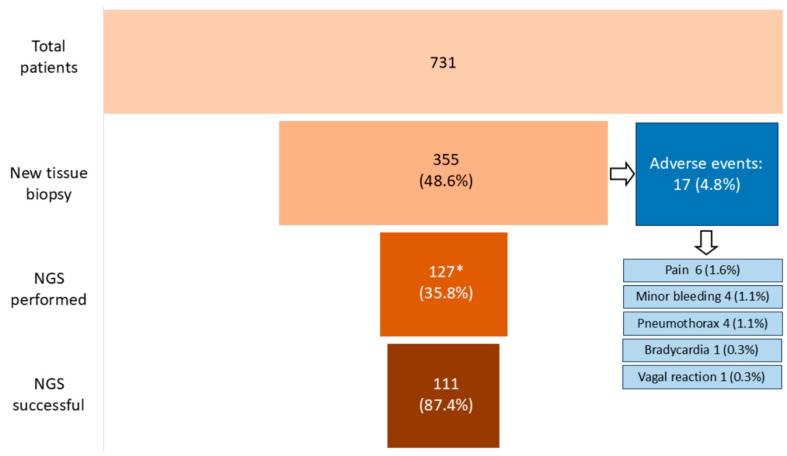
Schematization of study population and adverse events associated with biopsies. * Biopsies for which NGS results are available.

Seventeen patients (4.8%) experienced procedural complications during the biopsy, including six cases of pain, four instances of minor bleeding, four pneumothoraxes, one case of bradycardia, one occurrence of subcutaneous emphysema, and one vagal crisis. However, none of these complications required hospitalization or resulted in lasting effects, as all were graded as G1 or G2 according to the CTCAE v5. Treatment measures included painkillers for managing pain, atropine for addressing vasovagal reactions, and topical tranexamic acid for a patient experiencing G2 bleeding following a skin biopsy. All pneumothoraxes occurred as a result of lung biopsies. Patients receiving anticoagulant or antiplatelet medications (n = 47) were typically required to temporarily discontinue such treatments before undergoing biopsy, in accordance with current risk-based guidelines and specific drug labeling. Notably, none of these patients experienced bleeding or other adverse events related to the procedure. Furthermore, our analysis revealed no correlation between needle size and the occurrence of complications (*p* = 0.8). Figure 2 provides a graphical representation of the study population, illustrating adverse events and the overall success rates of NGS.

## 4. Discussion

Evaluating a drug’s impact on specific molecular targets in tumor tissue and identifying biomarkers within tumor cells that might correlate with response or resistance have become increasingly crucial for understanding cancer biology and discovering new drugs. Consequently, the utilization of optional or mandatory research biopsies, as well as the ethical and safety considerations associated with these procedures, plays a significant role in the early stages of clinical trial development. Our retrospective study highlights the frequent requirement of biopsies for enrollment in clinical trials, with nearly 50% of patients undergoing this procedure. This rate may still represent an underestimation, as in our clinical practice we routinely evaluate and discuss the opportunity for study biopsies based on the ASCO risk framework for study biopsies, and systematically request waivers for invasive procedures that pose the highest risk to patients. This approach aligns with the European Society for Medical Oncology (ESMO) Clinical Research Observatory recommendation to implement study procedures that do not compromise the best care for patients, especially when protocol requirements may be overly invasive.

In our cohort, the most common sites of biopsy were the liver, lymph nodes, skin, and breast—locations where metastases frequently occur and that are easily accessible.

Our study reports that research biopsies are safe in this setting, with a complication rate of less than 5%, similar to studies conducted in other patient populations. The observed 4.8% risk of non-severe complications aligns with the risk associated with diagnostic biopsy routinely performed in clinical practice [6].

The histological material obtained from biopsies in our cohort of patients was adequate for histological diagnosis in the vast majority of cases (98%), consistent with findings from previous studies [20,21].

For example, Lee et al. reported comparable results in patients with cancer undergoing research biopsies for clinical trials in Korea [22]. Among the 122 biopsies included in the study, the rate of adequacy for pathological diagnosis was 93.4%, while the success rate for molecular testing was 89.5%. However, the molecular testing success rate included various techniques, such as IHC, ISH, PCR, RNA sequencing, T-cell receptor sequencing, and only 14 NGS tests with unspecified characteristics, all of which were successful [22]. Adverse events occurred in 12 procedures (9.8%), all of which were mild [22]. Similarly, a retrospective study on the safety of needle biopsy in patients with neuroblastoma reported minor complications in 7 of 95 patients and one major complication, yielding a complication rate of 8.4% [20]. A prospective study in adults and children with hepatitis B reported 32 complications among 488 percutaneous liver biopsies (6.5%), including 7 serious adverse events [23]. Another study on percutaneous liver biopsies for NGS testing performed with puncture tract embolization reported a bleeding rate of 4.2% (4/96), with one major bleeding event requiring a blood transfusion [24].

Overall, our study confirms the safety of performing new biopsies in patients with advanced cancer, providing clinicians with a foundation to inform patients about the risks and benefits of the procedures when discussing enrollment in clinical trials that require biopsies. However, it is important to note that biopsy quality and complication rates, particularly for US-guided liver biopsies, are highly operator-dependent. Our study was conducted in a comprehensive cancer center, where imaging-assisted biopsies are performed by experienced interventional radiologists with extensive expertise. The standard procedure implemented by interventional radiologists at the European Institute of Oncology for percutaneous liver biopsies involves retrieving, whenever possible, three frustules with a single puncture of the liver capsule to reduce the risk of bleeding. It has been observed that multiple punctures of the liver capsule are associated with a higher risk of bleeding, albeit usually minor [25,26]. Specifically, evidence indicates a correlation between more than two passes and non-severe complications [27]. However, prior studies suggest that while operator experience affects the adequacy of liver tissue for pathology assessment, it does not influence the complication rate [26]. Our study also confirms that the needle size is not correlated with the occurrence of adverse events, consistent with previous reports [26].

Concerning NGS testing, our results indicate that a significant percentage of samples are inadequate for analysis, which can negatively impact patients by subjecting them to non-informative cancer sampling and, in some cases, excluding them from clinical trials, thereby missing the opportunity to identify actionable molecular alterations. Potentially actionable molecular alterations include agnostic targets (*NTRK1-2-3* [15], *RET* fusions [28], *BRAF* V600E mutations [29], microsatellite instability [30], high tumor mutational burden [31]), or tissue-specific targets, such as *EGFR* mutation in NSCLC [18] or *PIK3CA* mutations in breast cancer [32]. Interestingly, different tests have varying requirements for sample quantity and quality. In our experience, outsourced tests tend to have stricter requirements compared to in-house testing This discrepancy likely explains the higher failure rate observed with FoundationOne™ tests compared to GerSom tests in our cohort. Although both are targeted sequencing tests, they rely on different methods: Foundation One™ CDx is a hybrid-capture assay [33], while GerSom is a custom amplicon-based panel [34]. See Table 2 for a comparison of the requirements of these two tests.

From this comparison, it appears that the Foundation One™ CDx assay has higher standard requirements for tissue quantity. This may explain the higher inadequacy rate for Foundation One™ compared to GerSom.

In a study published in 2019, Eso et al. reported that the success rate of NGS testing on fresh tissue was 97.4% (38/39), with a 100% success rate on US-guided fresh liver biopsy samples (22/22) compared to 84.8% for archival FFPE tissue. The difference between fresh and archival samples was statistically significant (*p* < 0.05) [35]. However, only three patients in that study were tested with a Foundation One™ assay, making it difficult to compare those results with our study.

Accurate and shared guidelines for developing and validating NGS assays already exist [36,37], but it is important to implement specific Standard Operating Procedures for samples intended for NGS to improve the rate of successful testing, as proposed by Compton et al. [38]. Effective communication and cooperation among multidisciplinary team members are also fundamental for optimizing tissue acquisition procedures and ensuring that adequate samples are obtained for molecular testing.

The future implications of our study are significant for both clinical research and personalized medicine. First, the overall safety of biopsies in early-phase clinical trials, with a low rate of adverse events (4.8%), suggests that these procedures can be safely implemented more broadly. This could encourage greater patient participation, thereby facilitating the development of new therapies and expanding the use of molecular profiling to guide treatment decisions. However, the notable NGS failure rate (12.6%) presents challenges for the reliability of molecular diagnostics in both research and clinical practice. Identifying the root causes of these failures is critical for improving test accuracy and consistency. Factors such as tissue quality, biopsy technique, and specific limitations of NGS platforms may contribute to these failures. Future research should focus on optimizing biopsy procedures and refining sequencing technologies to minimize test failures. Addressing these issues will enhance the implementation of precision medicine, ensuring that patients receive the most appropriate therapies based on their genomic profile. Additionally, our findings have important implications for regulatory standards and guidelines related to biopsy procedures and NGS testing in clinical trials. As NGS becomes an increasingly integral part of cancer research and treatment, ensuring the reliability and safety of these processes will be crucial for advancing the field.

## 5. Conclusions

Approximately half of the patients referred to our EDD Unit underwent new biopsies. Overall, the procedure was safe, with only a small percentage of patients (4.8%) experiencing non-serious complications. Notably, around 10% of NGS attempts were unsuccessful due to inadequate sample quality or quantity. This underscores the critical need for implementing specific guidelines and Standard Operating Procedures for samples intended for NGS. By using a risk-based biopsy framework to guide clinical decisions, procedure-related complications may be minimized, while ensuring the collection of high-quality samples, to support drug development and advance precision medicine.

## Figures and Tables

**Table 1:** Characteristics of patients who underwent tissue biopsies. LMWH: Low-Molecular-Weight Heparin; NOAC: New Oral Anticoagulant.

Characteristics	Number of Patients n (%)	
Gender	Female, 280 (79%)	
	Male, 75 (21%)	
Median age	56 years (range: 20–83 years)	
Tumor type	Breast cancer, 190 (53.5%) Skin cutaneous melanoma, 24 (6.8%) Carcinoma of unknown primary, 21 (5.8%) Lung adenocarcinoma, 15 (4.2%) Cholangiocarcinoma, 14 (3.9%) Stomach adenocarcinoma, 12 (3.4%) Ovarian cancer, 12 (3.4%) Head and neck squamous cell carcinoma, 11 (3.1%) Mesothelioma, 7 (2.0%) Pancreatic adenocarcinoma, 6 (1.7%) Colorectal adenocarcinoma, 5 (1.4%) Urothelial cancer, 4 (1.1%) Lung squamous cell carcinoma, 4 (1.1%) Hepatocellular carcinoma, 3 (0.8%) Endometrial carcinoma, 3 (0.8%) Cervical squamous cell carcinoma, 2 (0.6%) Sarcoma, 1 (0.3%) Miscellanea, 21 (5.9%)	
Anticoagulant or antiplatelet therapy	Anticoagulant agents, 29 (8.2%)	LMWH, 27 (7.6%)
		NOAC, 2 (0.6%)
	Antiplatelets agents, 18 (5.1%)	
	No anticoagulant nor antiplatelets agents, 308 (86.7%)	

**Table 2:** Comparison of Foundation One™ CDx and GerSom assays. H&E: Hematoxylin and Eosin. USS: unstained slides. TN: tumor nuclei.

Test	Foundation One™ CDx [33]	GerSom [34]
FFPE	Allowed	Allowed
Decalcification	Not allowed	Unspecified
Sample size	Block + 1 H&E slide or 10 USS (5 µm thickness) + 1 H&E	Block or 3 USS (5 µm thickness)
Surface area or volume	25 mm^2^ (block) or 1 mm^3^ (slides)	Unspecified
Tumor content	Optimum: >30% TN Minimum: 20% TN	Unspecified
Number of genes tested	324	467

## Data Availability

Data are available upon request to corresponding author.
